# Physicochemical Properties of Pre-Treatment Tartary Buckwheat Flour and Its Effect on Dough and Noodle Quality

**DOI:** 10.3390/foods15111975

**Published:** 2026-06-02

**Authors:** Shengnan Xu, Xueqin Li, Jiayin Lv, Jie Chen, Kunlun Liu

**Affiliations:** 1 College of Food Science and Technology, Henan University of Technology, Zhengzhou 450001, China; 18861997009@163.com (S.X.); jeanlv0907@163.com (J.L.); cjie06@163.com (J.C.); knlnliu@126.com (K.L.); 2 Henan Province Wheat−Flour Staple Food Engineering Technology Research Centre, Zhengzhou 450001, China

**Keywords:** Tartary buckwheat, microwave pre-treatment, extrusion, rheological properties, textural characteristics

## Abstract

This study compared physical and chemical properties of extruded Tartary buckwheat flour (ETBF) and microwave-extruded Tartary buckwheat flour (M-ETBF), as well as the quality of dough and noodles made with these flours. The results showed that M-ETBF had higher protein and flavonoid contents, smaller particle size and darker color (lower L* value) compared to ETBF. With increasing ETBF or M-ETBF in wheat flour, gelatinization temperature rose and viscosity decreased. The dough became darker in color, with a lighter green and deeper yellow. Dough with 20% ETBF or M-ETBF had rheological properties similar to pure wheat dough. In noodles, increasing Tartary buckwheat flour decreased springiness, but also increased hardness, chewiness and cooking loss. The best taste and overall quality were at 20% addition for both types of flour. When added in equal amounts, M-ETBF noodles had slightly better viscosity and springiness.

## 1. Introduction

Tartary buckwheat (*Fagopyrum tataricum*) is a highly nutritious grain that contains significantly higher levels of rutin and other antioxidants than common buckwheat (*Fagopyrum esculentum*). It is also rich in essential trace minerals and dietary fiber, conferring multiple health benefits [1], including anti-carcinogenic effects, blood lipid regulation, and blood pressure stabilization [2,3]. In traditional Chinese medicine (TCM), Tartary buckwheat has long been recognized as a medicinal food, commonly used to tonify qi and blood and relieve fatigue. It also exhibits potential in alleviating rheumatic symptoms such as joint pain [4]. Owing to these distinctive health-promoting attributes, Tartary buckwheat has gained increasing consumer popularity and become a research hotspot in the cereal science field [5]. Cereal-based staple products, such as noodles, steamed buns, and rice noodles, play a pivotal role in Chinese dietary culture. Tartary buckwheat, with high nutritional density and unique health-promoting bioactive components, meets the growing demand for healthy diets. Its application in cereal-based staple products has received widespread attention in recent years.

However, preparing dough from Tartary buckwheat presents certain challenges, since it is gluten-free and lacks elasticity and extensibility, making it difficult to shape [6,7]. When exposed to water, rutin in Tartary buckwheat is quickly broken down by enzymes into quercetin and rutinose, which reduces its edible quality [8]. These issues limit Tartary buckwheat’s use in noodle production.

Pre-treating starch can help it swell quickly in cold water, stick better to gluten structures, and improve dough flexibility. Currently, many methods such as extrusion, microwaving, and baking are used to pre-treat Tartary buckwheat flour and improve its processing qualities. Extrusion pre-treatment especially helps by enhancing starch gelatinization, increasing water absorption, and improving gel formation. Studies have shown that extrusion increases the gelatinization degree, promotes the formation of a uniform structure in the dough, and improves its overall elasticity [9]. It also changes the starch and protein, leading to better cooking, texture, and the ability to add more Tartary buckwheat flour in products like noodles. Compared to untreated flour, extruded Tartary buckwheat flour shows significantly better gelatinization, hydration, and expansion properties, with improved protein cross-linking, making noodles more desirable [10]. Furthermore, appropriate thermal treatment has been demonstrated to effectively increase the flavonoid content in Tartary buckwheat flour and enhance its antioxidant capacity [11,12]. Noodles made from a blend of extrusion-modified buckwheat flour and wheat flour exhibit lower viscosity compared to traditional wheat noodles. However, these noodles had better oxidation resistance, and they were significantly harder, more elastic, and chewier. This shows that adding extruded buckwheat flour can improve both the textural quality and nutritional value of noodles. Extruded buckwheat flour also acts like an adhesive. It facilitates the binding of starch granules with other ingredients, thereby producing noodles that possess excellent cooking qualities, a pleasant texture, and sensory appeal [13]. Additionally, during extrusion, the high temperature and pressure deactivate enzymes in Tartary buckwheat, which helps prevent the breakdown of rutin. However, crushing the grains into flour can still reduce rutin content. Some studies have shown that heat treatments like microwave, extrusion, cooking, or baking can modify buckwheat flour for noodle-making [14,15]. But, so far, no research has combined microwave and extrusion methods for Tartary buckwheat flour. In this study, we compared extruded Tartary buckwheat flour (ETBF) and microwave-extruded Tartary buckwheat flour (M-ETBF). On this basis, an exploration will be conducted into the impacts of ETBF or M-ETBF with different addition ratios on the physicochemical properties of dough or noodles, thereby establishing a comprehensive research chain spanning from raw material processing to product quality.

## 2. Materials and Methods

### 2.1. Materials

Tartary buckwheat seeds were purchased from Liangshan, Sichuan Province. They were selected to be full, uniform in size, with intact and unbroken seed shells, and free from pests and diseases. Wheat flour was purchased from Zhengzhou Jinyuan Noodle Co. (Zhengzhou, China), and salt from Henan Salt Industry Group Co. (Zhengzhou, China).

### 2.2. Determination of Basic Nutrients

The moisture, ash, protein, starch and fat in Tartary buckwheat were measured using standard methods (GB 5009.3-2016, GB 5009.4-2016, GB 5009.5-2016, GB 5009.9-2023 and GB 5009.6-2016) [16,17,18,19,20]. The extraction and determination of total flavonoids were carried out following the method described in NY/T 1295-2007 [21].

#### 2.2.1. Preparation of Test Sample Solutions

In total, 0.2 g of the sample (1 g for Tartary buckwheat products, all weighed to the nearest 0.0001 g) was accurately weighed into a 50 mL conical flask. Then, 30 mL of 70% (*v*/*v*) aqueous methanol solution was precisely pipetted into the flask. The mixture was incubated with shaking for 2 h in a constant-temperature water bath shaker (SHA-CA, Changzhou Guowang Instrument Manufacturing Co., Ltd, Changzhou, China) maintained at 65 ± 2 °C. After filtration, the filtrate was made up to a final volume of 50 mL with 70% (*v*/*v*) aqueous methanol and stored at 4 °C until subsequent analysis.

#### 2.2.2. Determination of Total Flavonoid Content

- Standard Curve Construction

Aliquots of 0.25, 0.50, 1.00, 2.00, 3.00 and 4.00 mL of 0.05 mg/mL rutin standard solution were pipetted into separate 10 mL volumetric flasks. To each flask, 2 mL of 0.1 mol/L AlCl_3_ solution and 3 mL of 1 mol/L CH_3_COOK solution were added. The solution was diluted with methanol and mixed well. A reagent blank was prepared in parallel without rutin. After standing at room temperature for 30 min, absorbance was measured at 420 nm.

The standard curve was Y = 23.066x − 0.001 (R^2^ = 0.9999), where x is rutin concentration (mg/mL) and Y is absorbance at 420 nm. Total flavonoid content was calculated from the standard curve.

B. Determination of Total Flavonoid Content in Samples

In total, 1 mL of the test sample solution was accurately pipetted into a 10 mL volumetric flask, and absorbance was measured using the identical procedure as described in Section A. The total flavonoid content was calculated according to the following formula:

$$\mathrm{Total}\;\mathrm{flavonoid}\;\mathrm{content}\;(\mathrm{mg}\;\mathrm{RE}/g)\;=\;\frac{C\;\times \;V\;\times \;D\;\times \;100}{m\;\times \;1000\;\times \;(100\;-\;H)}\times 100$$

Notes: *C*, total flavonoid concentration of the test solution derived from the standard curve, mg/mL; *V*, volume of the test solution, mL; *D*, total dilution factor of the sample; *m*, mass of the sample, g; H, moisture content of the sample, % (mass fraction).

### 2.3. Pre-Treatment of Tartary Buckwheat Seeds

#### 2.3.1. Preparation of ETBF

The seeds were crushed into flour for extrusion processing. The process parameters were: feed rate of 4.14 kg/h, material moisture content of 22%, and temperature zones of 40 °C, 130 °C, and 170 °C. The extruded material was then crushed and sieved through an 80-mesh screen.

#### 2.3.2. Preparation of M-ETBF

To prepare pre-treated seeds, 34 g of water was added to 100 g of hulled Tartary buckwheat seeds, followed by a 14 h soaking period until the moisture content reached 47.75%. After soaking, the seeds were microwaved for 4 min at medium heat in a 150 mm diameter dish. Once cooled to room temperature, they were crushed and extruded using the same conditions as ETBF. The extruded material was then crushed and sieved through an 80-mesh screen.

### 2.4. Determining Gelatinization Characteristics

The gelatinization characteristics of the mixed flour were determined using the Viscograph-E rapid viscosity analyzer from Brabender Corporation, Duisburg, Germany. Based on the method proposed by Cheng et al. [22], an optimized measurement of gelatinization indicators was carried out. All indicators were set with three parallel experiments.

### 2.5. Determination of Color

The color parameters were determined according to the method described by Zhang et al. [23] with minor modifications. A colorimeter (WAC-S, Shanghai Yidian Physical & Optical Instrument Co., Ltd., Shanghai, China) was preheated for 30 min and calibrated with a standard white tile before testing. For each sample, three parallel measurements were performed at different positions, and the average values of L* (lightness), a* (red–green value), and b* (yellow–blue value) were recorded.

### 2.6. Determination of Tartary Buckwheat Flour Particle Size

The flour sample (0.6 g) was mixed with deionized water (12 mL), subjected to ultrasonic treatment for 30 min, and stirred until a smooth surface was obtained. Particle size was measured using a laser particle size analyzer (WAC-S, Shanghai Yidian Physical & Optical Instrument Co., Shanghai, China) within a range of 0.1–5000 nm. Each measurement was performed in triplicate.

### 2.7. Determination of Microstructure of Tartary Buckwheat Flour

A small amount of flour was evenly spread on double-sided adhesive tape attached to the carrier stage, with different samples prepared using various gelatinization techniques. After gold-coating with a gold-plating device, observations were conducted employing a scanning electron microscope (su8010, Hitachi High-Tech Corporation, Tokyo, Japan) set at a magnification of 500×.

### 2.8. Measuring Thermal Properties of Mixed Flours

The thermal properties of the composite flour were characterized by differential scanning calorimeter (TAQ20, TA Instruments, New Castle, DE, USA), following He et al. [24]. We weighed 2.5 mg of mixed Tartary buckwheat flour into a DSC crucible, then added 7.5 μL of distilled water. The mixture was rested for 24 h before testing. The heating rate is set at 10 °C/min, with the temperature increasing from 20 °C to 120 °C, and the nitrogen flow rate is set at 20 mL/min.

### 2.9. Testing Kneading and Mixing Properties

Dough kneading and mixing properties were determined using a 10 g constant-weight mixograph (National Manufacturing, Lincoln, NE, USA) following AACC Approved Method 54-40.01 [25]. Flour samples were corrected to a 14% moisture basis and weighed accurately. The mixing bowl was pre-equilibrated to 30 °C prior to testing. Distilled water (25 °C) was added, and the dough was mixed until the mixogram peak was attained, followed by an additional 2 min of mixing and then data was recorded.

### 2.10. Determination of Dynamic Rheology of Mixed Doughs

Dynamic rheological measurements were performed using a rotational rheometer (505SS, TA Instruments, New Castle, DE, USA) with a 40 mm parallel plate geometry (2 mm gap) at 25 °C, according to Gao et al. [26] with slight modifications. The linear viscoelastic region was identified via strain sweep (0.01–10%), and frequency sweeps were conducted at 0.1% strain over 0.1–20 Hz.

### 2.11. Preparation of Tartary Buckwheat Noodles

Wheat flour was blended with ETBF and M-ETBF at mass substitution levels of 0%, 10%, 20%, 30%, and 40% (w/w) for subsequent processing. The composite flour was transferred into a dough mixer (JHMZ-200, Beijing Dongfu Jiuheng Instrument Technology Co., Ltd., Beijing, China) and premixed for 4 min. Saline solution was subsequently added, and mixing was continued for an additional 4 min until a homogeneous dough was obtained. The resulting dough was proofed in a constant-temperature incubator (SPX-150-II, Shanghai Yuejin Medical Device Co., Ltd., Shanghai, China) at 25 °C for 15 min. Following proofing, the dough was rolled into a 1 mm thick sheet and cut into uniform noodle strips measuring 20 cm in length and 1 mm in width. The obtained fresh noodles were used for the subsequent measurement of indicators.

### 2.12. Determination of Cooking Quality of Tartary Buckwheat Noodles

A total of 500 mL of distilled water was precisely measured into a beaker and heated to boiling. Then, 10 g of fresh wet noodles (W_0_) was weighed, cooked in the boiling water, removed, and cooled in deionized water for 1 min. After draining for 1 min and blotting dry with filter paper, the noodle mass was recorded as W_1_. All cooking water was transferred to a 500 mL volumetric flask, diluted to the mark with deionized water, and mixed thoroughly by repeated inversion. A 50 mL aliquot of the cooking water was transferred to a pre-weighed constant-weight beaker (W_2_). The aliquot was evaporated to near dryness on a hot plate, then oven-dried to constant weight. After cooling to room temperature, the total mass was recorded as W_3_. Calculations were performed using the formula below.

$$\mathrm{Cooking}\;\mathrm{loss}\;\mathrm{rate}\;(\%)=10\;\times \frac{\left(W_{3}-W_{2}\right)}{W_{0}}\;\times \;100$$

$$\mathrm{Water}\;\mathrm{absorption}\;(\%)=\frac{\left(W_{1}-W_{0}\right)}{W_{0}}\times 100$$

### 2.13. Determination of Texture Properties of Tartary Buckwheat Noodles

Textural properties of noodles were measured using a texture analyzer (TA-XT Plus, Stable Micro System, Lon, UK), with a slight modification to the testing method based on the study by Li et al. [27]. Parameter settings: HDP/PFS probe, test mode: compression, pre-test speed: 3.00 mm/s, in-test and post-test speed: 1.00 mm/s, compression level: 75%, time interval: 5 s, trigger force: 5 g, calibration distance: 25 mm.

### 2.14. Determination of Tensile Properties of Tartary Buckwheat Noodles

Tensile properties of noodles were measured using a physical tester with an A/KIE probe; the testing method was based on the research by Huiyu et al. [28]. Parameter settings: test mode: tension, pre-test speed: 2.00 mm/s, in-test speed: 2.00 mm/s, post-test speed: 10.00 mm/s, extension distance: 25.00 mm, trigger force: 5 g, calibration distance: 10 mm.

### 2.15. Determination of Sensory Properties of Tartary Buckwheat Noodles

Sensory panelists (*n* = 8, 4 males and 4 females) were selected via sensory acuity tests and professional training, and validated to conduct standardized and precise assessments of the sensory attributes of Tartary buckwheat noodles, including color, texture, and flavor. Noodle preparation for sensory evaluation: Fresh noodles were cooked in boiling water until reaching the optimum cooking time (defined as the time when the white core of the noodle completely disappeared upon pinching between two fingers). The cooked noodles were immediately removed from the boiling water, rinsed briefly with cold running water, plated, and promptly presented to the trained sensory panel for evaluation. The sensory evaluation criteria (parameters and scoring scales) are presented in Table 1.

### 2.16. Statistical Analysis

Each experiment was performed in triplicate, and data were expressed as mean ± standard deviation (SD). One-way ANOVA followed by Duncan’s multiple range test was used to determine significant differences between groups (*p* < 0.05). The results were analyzed using IBM SPSS version 23.0 (SPSS Inc., Chicago, IL, USA). Origin 8.0 software (OriginLab, Northampton, MA, USA) was used for plotting.

## 3. Results and Discussion

### 3.1. Effect of Pre-Treatment on the Properties of Tartary Buckwheat Flour

#### 3.1.1. Basic Physicochemical Properties

Table 2 shows the basic physicochemical properties of different Tartary buckwheat flours. The protein and total flavonoid content are higher in M-ETBF compared to ETBF. This means M-ETBF is more nutritious. ETBF is made by crushing the Tartary buckwheat grains and then extruding them after water treatment. During this process, enzymes that break down rutin contact rutin and quickly turn it into quercetin and rutinose, lowering the total flavonoids [29]. In contrast, M-ETBF is pre-treated with microwave water treatment before grinding. This hydrothermal process inhibits enzyme activity, helping prevent rutin from breaking down. The changes in protein and fat content in both flours are related to how fats, starches, and proteins form complexes during pre-treatment. Instant high temperature and strong shear during single extrusion severely damage cell structures. Bound fat is rapidly released in a short period and cannot form stable complexes with starch and protein, with most fat lost through water evaporation and screw shearing. Microwave pre-treatment mildly and gradually disrupts cell structures to slowly release bound fat. In subsequent extrusion, pre-released free fat fully interacts with starch and protein to form stable complexes, effectively reducing fat loss during processing [30,31].

#### 3.1.2. Color, Particle Size, and Microstructure

**Figure 1:**
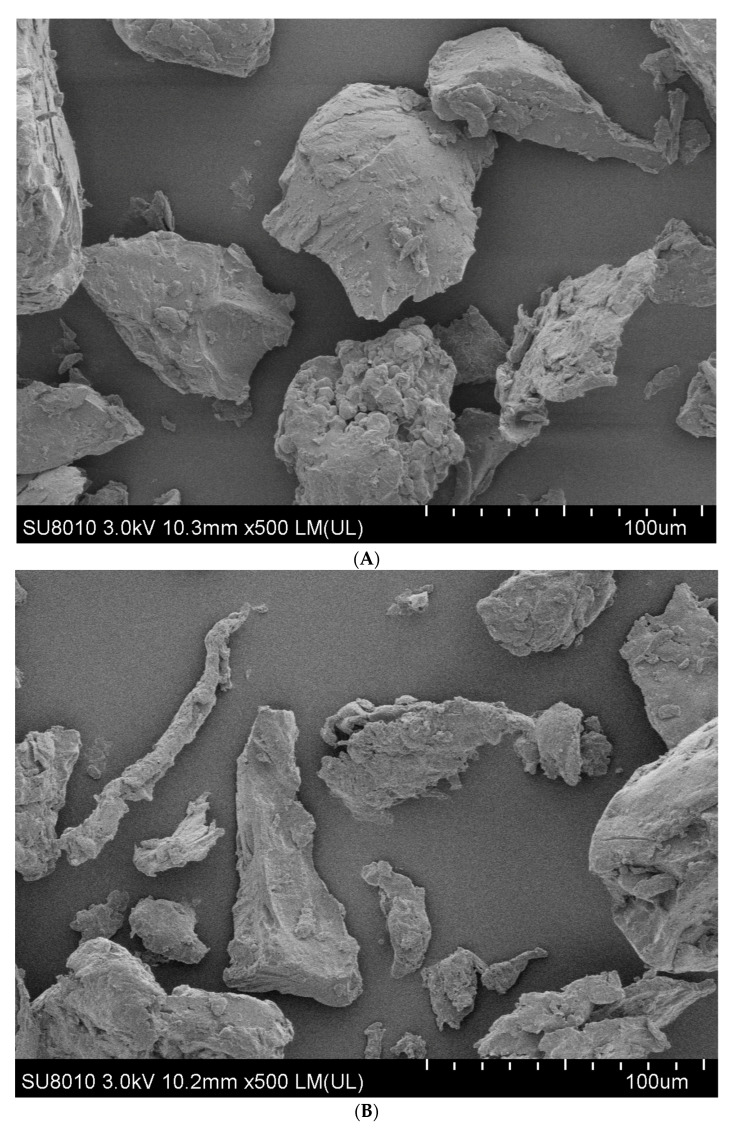
The microstructure of different Tartary buckwheat flours. Notes: (**A**) and (**B**) represent the microstructure of ETBF and M-ETBF magnified by 500 times, respectively.

The color of noodles impacts consumer appeal. Table 3 shows that M-ETBF is darker (lower L* value) than ETBF, likely because of higher flavonoid and polyphenol content, and changes from reactions like Maillard browning, starch gelatinization, and protein modifications under high heat and pressure [32,33,34]. Similar findings have been reported in the literature. Pre-gelatinization causes a decrease in lightness (L* value) and darker coloration of Tartary buckwheat flour. The extent of the Maillard reaction during extrusion is strongly correlated with the pre-treatment method of the raw material. Microwave pre-treatment significantly enhances the formation of Maillard reaction products (MRPs), which consequently reduces the lightness (L* value). This conclusion is also applicable to other crops including soybeans and corn [35]. Particle size affects the flour’s structure and nutrition. M-ETBF’s particles are smaller than ETBF’s, because microwave treatment causes polar molecules to vibrate rapidly, breaking down particles. Figure 1 shows the microstructures: both types have large, irregular particles with rough surfaces, tiny holes, and indentations. M-ETBF particles are smaller, consistent with the reduced particle size observed.

### 3.2. Effect of Pre-Treatment Tartary Buckwheat Flour on Dough Quality

#### 3.2.1. Color

**Figure 2:**
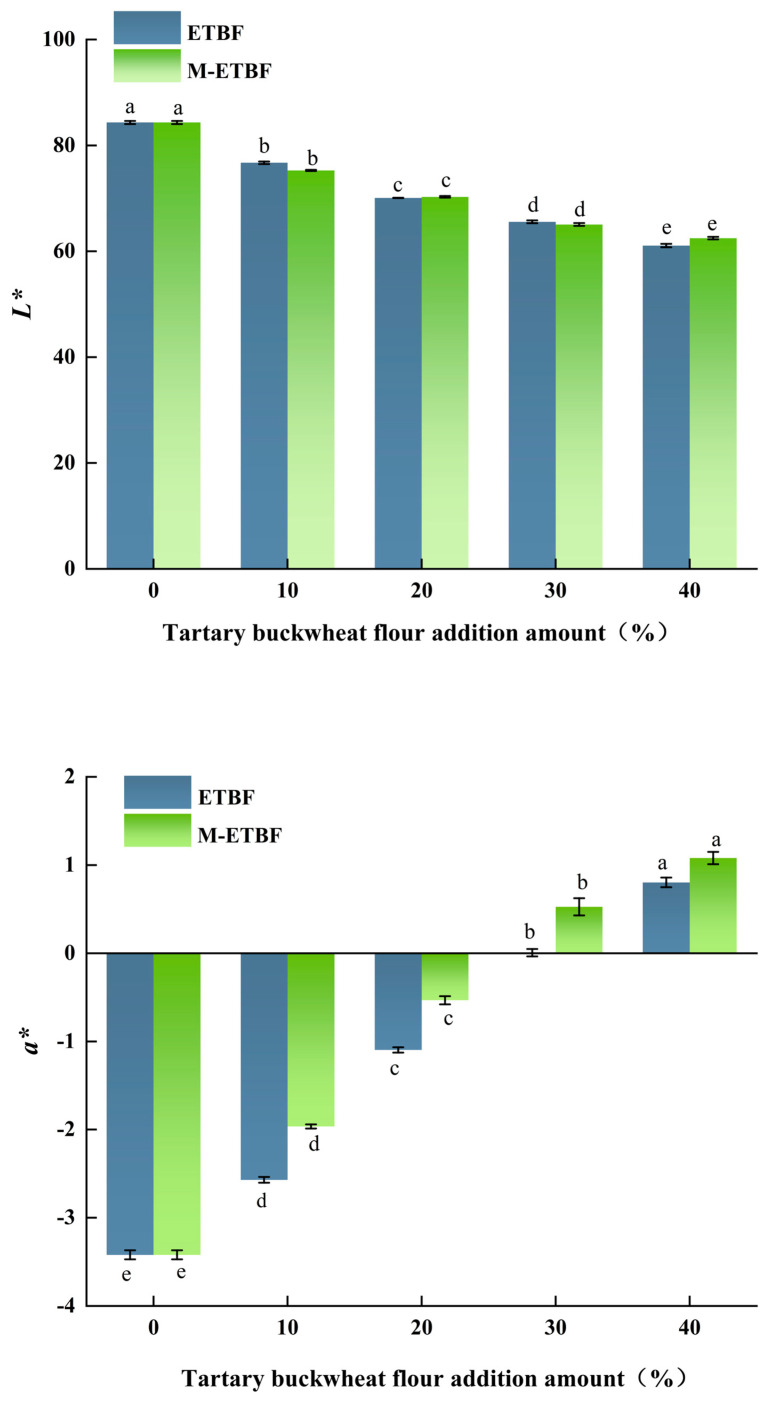

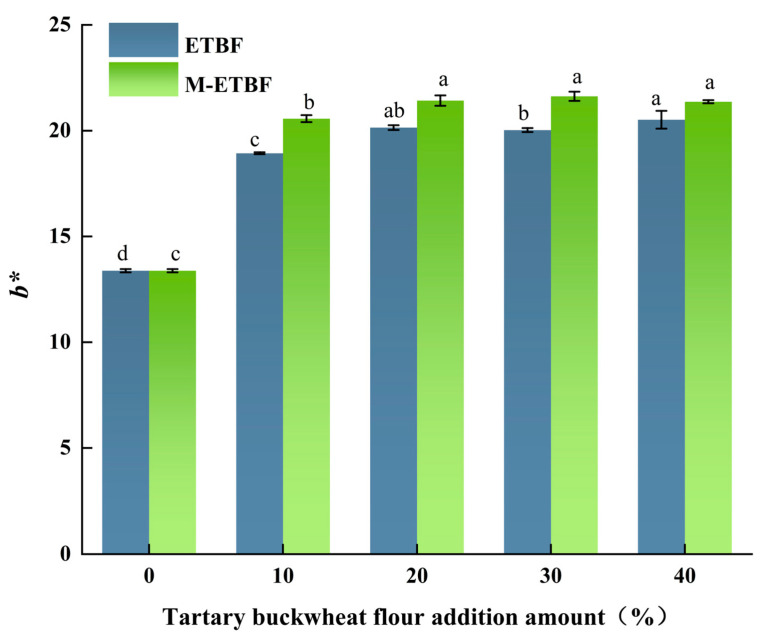
Effect of pre-treatment Tartary buckwheat flour on the color of dough. Notes: L*, lightness; a*, red–green value; b*, yellow–blue value. Different letters indicate significant differences between different samples (*p* < 0.05).

Figure 2 illustrates the effects of Tartary buckwheat flour with different pre-treatments on dough color. The L* value of dough decreased significantly with the increasing addition of ETBF and M-ETBF, indicating the darkening of dough color, which was attributed to the accumulation of Maillard reaction products. Notably, lower L* values were observed in dough supplemented with M-ETBF. At the same addition level, higher a* and b* values were detected in dough prepared with M-ETBF rather than ETBF. This was because microwave-assisted extrusion facilitated the release of total flavonoids and accelerated the Maillard reaction as well as polyphenol liberation, which is consistent with previous findings. Owing to the darker inherent color of M-ETBF modified by microwave extrusion, the corresponding dough presented a darker appearance.

#### 3.2.2. Thermal Properties

The onset temperature (T_0_) indicates how easily the starch begins to gelatinize: a lower T0 means water can access and gelatinize the starch more easily. The gelatinization enthalpy (ΔH) is the energy required to gelatinize the starch. Higher starch content requires more energy [36]. Table 4 shows the thermal properties of blends with different addition levels of ETBF and M-ETBF. As more ETBF or M-ETBF was added, both the onset temperature (T_0_) and peak temperature (T_p_) of gelatinization increased significantly, while ΔH decreased significantly. This suggests that adding these flours delays gelatinization: it takes higher temperatures, and less energy is needed to gelatinize the starch. This is because Tartary buckwheat has more dietary fiber than wheat, which competes for water during heating, making it harder for starch to gelatinize and increasing the required temperature. The decrease in ΔH shows that more heat is needed to break down the crystal structure of the starch. When equal amounts of ETBF and M-ETBF are added, there is no significant difference in T_0_, T_p_ or ΔH. Vicente et al. [31] demonstrated that microwave hydrothermal treatment decreased the gelatinization enthalpy and increased the peak gelatinization temperature (T_p_) of Tartary buckwheat flour by 3.7 °C. Zhang et al. [37] found that twin-screw extrusion at 120 °C reduced the gelatinization enthalpy of Tartary buckwheat flour by 93.7%, and most of the ordered starch structures were disrupted. Both are consistent with the trend of this study.

**Table 4:** Effect of pre-treatment Tartary buckwheat flour on the thermal properties of flour.

Samples	Addition Amount/%	T_0_/°C	T_p_/°C	T_c_/°C	ΔH/(J/g)
ETBF	0	56.66 ± 0.20 ^b^	62.09 ± 0.12 ^c^	76.37 ± 0.11 ^a^	6.71 ± 0.06 ^a^
	10	59.43 ± 0.54 ^a^	62.89 ± 0.07 ^b^	77.41 ± 0.59 ^a^	6.29 ± 0.07 ^b^
	20	58.18 ± 0.67 ^ab^	63.09 ± 0.04 ^b^	76.04 ± 0.15 ^ab^	5.21 ± 0.03 ^c^
	30	58.10 ± 0.12 ^ab^	63.10 ± 0.09 ^b^	74.69 ± 0.37 ^bc^	4.58 ± 0.22 ^d^
	40	58.75 ± 0.25 ^a^	63.55 ± 0.09 ^a^	73.68 ± 0.45 ^c^	4.09 ± 0.04 ^e^
M-ETBF	0	56.66 ± 0.20 ^b^	62.09 ± 0.12 ^d^	76.37 ± 0.11 ^a^	6.71 ± 0.06 ^a^
	10	57.02 ± 0.02 ^b^	63.19 ± 0.07 ^c^	77.52 ± 0.64 ^a^	6.54 ± 0.11 ^a^
	20	58.37 ± 0.05 ^a^	63.92 ± 0.03 ^ab^	75.89 ± 0.16 ^a^	5.58 ± 0.17 ^b^
	30	58.71 ± 0.04 ^a^	63.77 ± 0.08 ^b^	76.10 ± 0.61 ^a^	5.12 ± 0.10 ^c^
	40	58.35 ± 0.16 ^a^	64.09 ± 0.02 ^a^	77.74 ± 0.64 ^a^	3.78 ± 0.14 ^d^

Notes: T_0_, onset temperature; T_p_, peak temperature; T_c_, final temperature; ΔH, gelatinization enthalpy; ETBF, extruded Tartary buckwheat flour; M-ETBF, microwave-extruded Tartary buckwheat flour. Different letters in the same column indicate significant differences (*p* < 0.05).

#### 3.2.3. Gelatinization Properties

Table 5 shows the gelatinization properties of blends with different addition levels of ETBF and M-ETBF. As more ETBF or M-ETBF was added, the peak, trough, and final viscosities of the mixed flour all decreased significantly. This means the flour’s ability to gelatinize was reduced. This happens because starch molecules become smaller after pre-treatment, breaking their molecular chains. When this occurs, the interactions between starch molecules weaken, leading to lower viscosity. Also, dietary fibers in ETBF and M-ETBF compete with wheat starch for water, which inhibits starch swelling and further lowers the viscosity. Breakdown viscosity (indicating thermal stability) and setback viscosity (related to starch retrogradation) also decreased with more additions of ETBF or M-ETBF. Smaller starch molecules do not easily form crystalline structures during cooling, making the starch less prone to hardening or retrogradation. When equal amounts of ETBF and M-ETBF were added, the viscosities of M-ETBF mixed flour were slightly higher, suggesting slight differences in their effects. In addition, microwave extrusion alleviates the destructive effect of Tartary buckwheat protein on the wheat gluten network, while simultaneously forming a more stable composite protein network through moderate cross-linking, thereby effectively balancing the processing properties and nutritional characteristics of dough; this is consistent with the findings of Jia et al. [38].

In-situ infrared spectroscopy has confirmed that during microwave heating, the vibrational intensity of O-H bonds changes prior to that of C-O-C bonds in starch-water systems. This indicates that energy is preferentially absorbed by polar water molecules and subsequently transferred to starch molecular chains, triggering progressive hydrogen bond cleavage and gelatinization. Consequently, the disruption of starch granule structure is less thorough than that achieved by conventional extrusion, thereby preserving certain inherent viscosity characteristics [39]. Zhang et al. [40] reported that after microwave pre-treatment of red rice at 640 W for 2 min, the RVA peak viscosity decreased from 2078.67 cP to 1245.33 cP, representing a mere 40.1% reduction. This value is significantly lower than the >80% viscosity reduction typically achieved by conventional extrusion processing, consistent with the mild disruption of starch granule structure induced by microwave treatment.

**Table 5:** Effect of pre-treatment Tartary buckwheat flour on the gelatinization properties of flour.

Samples	Addition Amount/%	PV/cP	TV/cP	BV/cP	FV/cP	SV/cP
ETBF	0	1253 ± 18 ^a^	531 ± 14 ^a^	721 ± 3 ^a^	1218 ± 19 ^a^	687 ± 5 ^a^
	10	911 ± 4 ^b^	386 ± 1 ^b^	525 ± 3 ^b^	933 ± 5 ^b^	547 ± 4 ^b^
	20	646 ± 15 ^c^	305 ± 7 ^c^	341 ± 7 ^c^	722 ± 14 ^c^	417 ± 7 ^c^
	30	477 ± 0 ^d^	260 ± 1 ^d^	216 ± 1 ^d^	567 ± 2 ^d^	307 ± 2 ^d^
	40	375 ± 24 ^e^	232 ± 12 ^d^	142 ± 11 ^e^	471 ± 25 ^e^	239 ± 13 ^e^
M- ETBF	0	1253 ± 18 ^a^	531 ± 14 ^a^	721 ± 4 ^a^	1218 ± 19 ^a^	687 ± 5 ^a^
	10	938 ± 1 ^b^	390 ± 2 ^b^	548 ± 2 ^b^	943 ± 2 ^b^	553 ± 0 ^b^
	20	685 ± 2 ^c^	320 ± 1 ^c^	365 ± 1 ^c^	752 ± 2 ^c^	432 ± 1 ^c^
	30	524 ± 6 ^d^	286 ± 5 ^d^	238 ± 1 ^d^	606 ± 5 ^d^	320 ± 1 ^d^
	40	407 ± 4 ^e^	253 ± 1 ^e^	153 ± 4 ^e^	513 ± 2 ^e^	259 ± 2 ^e^

Notes: PV, peak viscosity; TV, trough viscosity; BV, breakdown viscosity; FV, final viscosity; SV, setback viscosity; ETBF, extruded Tartary buckwheat flour; M-ETBF, microwave-extruded Tartary buckwheat flour. Different letters in the same column indicate significant differences (*p* < 0.05).

#### 3.2.4. Dough Kneading and Mixing

Table 6 shows how different pre-treated Tartary buckwheat flours affect dough kneading and mixing. Increasing ETBF reduces the time and area of the peak, mainly because ETBF decreases gluten content and friendly fibers damage gluten structure. This makes the dough less elastic and harder to knead. However, when M-ETBF was added at 20% or less, its impact on kneading time and peak area was small. The 8 min bandwidth (which indicates dough strength and viscosity) increased with small amounts of both flours, meaning the dough became stronger and more viscous. But at 40% and higher, the bandwidth decreased significantly, showing the dough became weaker and less cohesive and its network structure broke down. As more Tartary buckwheat flour was added, the slope on the right side increased, indicating the dough’s stability declined and kneading resistance decreased. When ETBF and M-ETBF were added equally, ETBF significantly shortened the dough formation time and peak curve area, while M-ETBF had a smaller effect at lower addition levels. Peng et al. [41] prepared buckwheat–wheat composite flours by incorporating different levels of buckwheat flour, and investigated their mixing properties, dough microstructure, and the sensory quality of resulting buckwheat steamed bread. The results showed that at buckwheat addition levels of 5–15%, no significant changes were observed in dough development time (DDT) and peak area, and the gluten protein network in the dough microstructure was only slightly weakened. However, when the addition level reached 20%, both DDT and peak area decreased markedly, accompanied by significant deterioration of the gluten network microstructure. These findings are consistent with the results obtained in the present study.

#### 3.2.5. Dynamic Rheology

**Figure 3:**
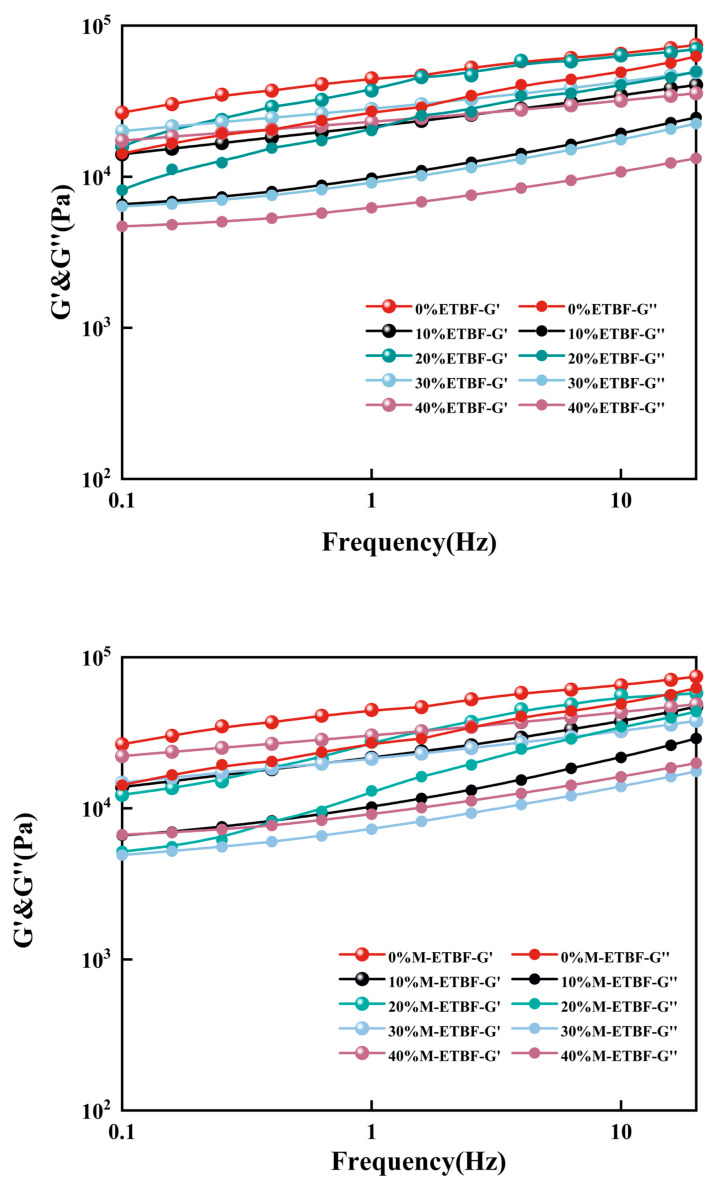

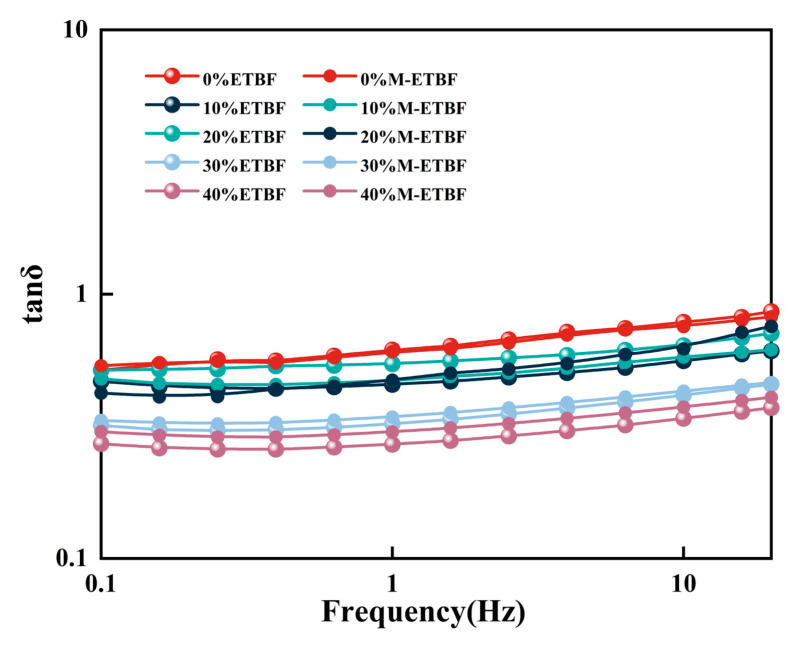
Effect of pre-treatment Tartary buckwheat flour on the dynamic rheological properties of dough. Notes: G′, elastic modulus; G″, viscous modulus; tanδ, the ratio of G″ to G′.

The elastic modulus (G′) and the viscous modulus (G″) are key parameters that describe how dough behaves. G′ shows how elastic or springy the dough is, while G″ is its viscosity or flow-ability [42]. Figure 3 displays how different pre-treated Tartary buckwheat flours influence dough’s rheological properties. The results show that both G′ and G″ increased for dough with ETBF and M-ETBF in the frequency range of 0.1–20 Hz. Throughout all samples, G′ was higher than G″, meaning the dough was mostly elastic and in a solid form [43]. Lisovska et al. [44] subjected Spanish and Polish buckwheat flours, as well as two teff flours (white teff and brown teff), to microwave-assisted hydrothermal treatment at an initial moisture content of 30%. The results demonstrated that the storage modulus (G′) of all treated samples increased significantly: by 32% and 16% for Polish and Spanish buckwheat flours, respectively, and by 14% and 18% for white and brown teff cultivars, respectively. This enhanced gel strength and structural stability were independent of species or cultivar differences, thereby significantly improving the formability and printability of the materials during 3D printing. When ETBF and M-ETBF were added at 20%, the rheological properties resembled those of wheat dough more closely. However, overall, dough with M-ETBF showed better viscoelasticity and was more similar to wheat dough than dough with ETBF.

### 3.3. Effect of Pre-Treatment Tartary Buckwheat Flour on Noodle Quality

#### 3.3.1. Cooking Quality

**Figure 4:**
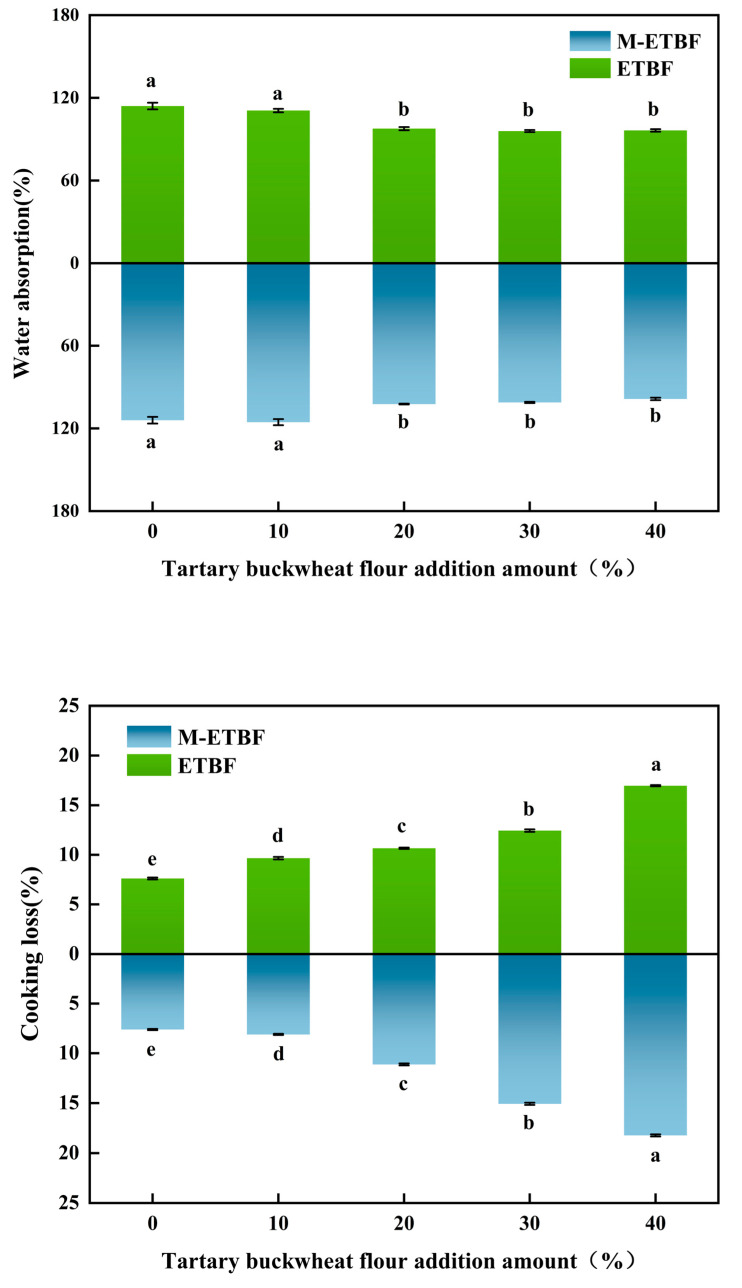
Effect of pre-treatment Tartary buckwheat flour on the cooking quality of noodles. Notes: Different letters indicate significant differences between different samples (*p* < 0.05).

Figure 4 compares the cooking qualities of noodles made with ETBF and M-ETBF. As the amount of Tartary buckwheat flour increased, both water absorption and cooking loss changed significantly: water absorption decreased, and cooking loss increased. Water absorption drops because the pre-treatment causes starch in Tartary buckwheat to gelatinize poorly and reduces its water-holding capacity. Adding Tartary buckwheat also lowers gluten protein content, weakening the noodle structure, which further reduces water absorption [45,46]. The higher cooking loss with Tartary buckwheat noodles occurs because extrusion breaks down starch and other large molecules into smaller ones, increasing water-soluble substances. Also, adding Tartary buckwheat may loosen the gluten network in noodles, allowing more soluble solids and starch particles to leak into the cooking water, thus increasing cooking loss [47]. At the same addition level, M-ETBF noodles had higher water absorption than ETBF noodles. When Tartary buckwheat flours were added at 10%, M-ETBF noodles also had lower cooking loss than ETBF. But when the addition was more than 20%, M-ETBF noodles showed higher cooking loss than ETBF noodles. Guo et al. [48] reported that moderately gelatinized buckwheat flour significantly improved the cooking quality and textural properties of noodles, yielding a breaking rate of only 10.11% at an addition level of 15%. This finding aligns with the results of the present study: noodles supplemented with 10% M-ETBF exhibited increased water absorption and no significant difference in cooking loss compared with the control group. However, with further increases in the addition levels of both ETBF and M-ETBF, the cooking loss of noodles increased significantly [49].

#### 3.3.2. Textural Quality

**Figure 5:**
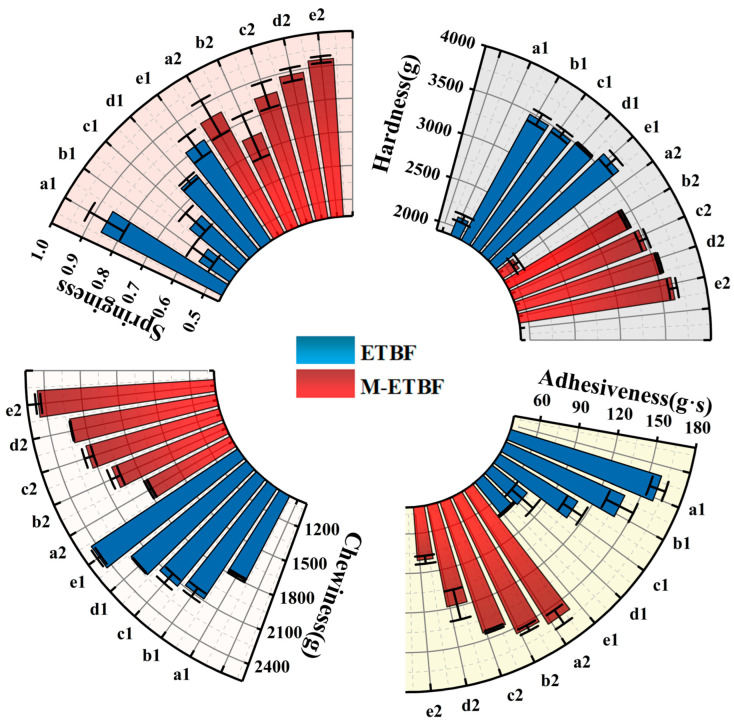
Effect of pre-treatment Tartary buckwheat flour on the textural quality of noodles. Notes: a1, b1, c1, d1, e1 correspond to 0%, 10%, 20%, 30%, and 40% of the ETBF addition amount respectively; a2, b2, c2, d2, e2 correspond to 0%, 10%, 20%, 30%, and 40% of the M-ETBF addition amount respectively.

Figure 5 shows the texture analysis of ETBF and M-ETBF noodles. As more of these flours were added, the noodles became harder and chewier. This happens because Tartary buckwheat flour reduces gluten protein, and since it absorbs less water than gluten, it results in tougher noodles. This also explains why water absorption decreased as more Tartary buckwheat was added. Adding Tartary buckwheat flour made the noodles less springy, but with higher amounts, springiness increased, and viscosity decreased. Pre-gelatinization treatment induces cracks and pores on the surface of Tartary buckwheat starch granules, increasing their contact area with water molecules. During cooking, these granules can rapidly absorb water to form a dense reticular gel structure, while promoting interactions between starch and wheat gluten proteins, thereby significantly enhancing the elasticity, chewiness and structural stability of noodles [50,51]. Overall, adding both M-ETBF and ETBF increased hardness and chewiness. However, M-ETBF noodles had slightly better adhesiveness and springiness than ETBF noodles at the same levels. M-ETBF noodles were harder, chewier, more springy, and had a fresher texture, which better meets consumers’ preferences for noodle texture.

#### 3.3.3. Tensile Quality

**Figure 6:**
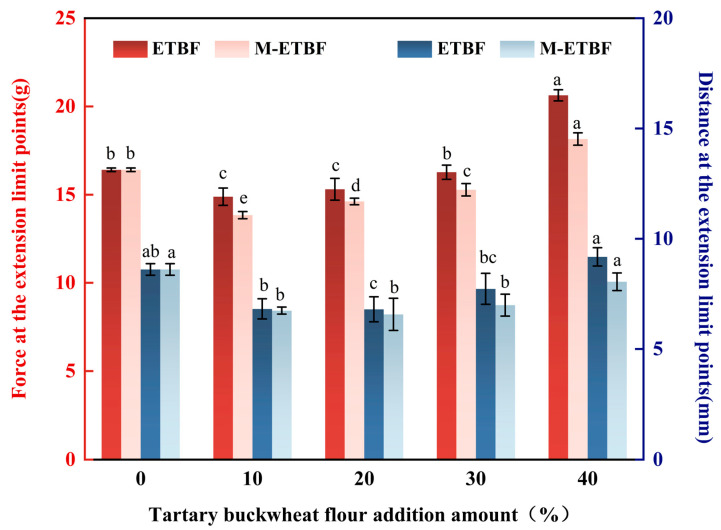
Effect of pre-treatment Tartary buckwheat flour on the extension quality of noodles. Notes: Different letters in the same column indicate significant differences (*p* < 0.05).

Figure 6 shows that the tensile properties of ETBF and M-ETBF noodles follow the same trend. Adding these flours decreased the noodles’ tensile force and stretchability at first. As the amount of flour increased, these properties then significantly improved. This improvement is related to the formation of a gel network from starch gelatinization in Tartary buckwheat flour. During cooking, the starch swells, pastes, and forms a continuous gel inside the noodles, helping compensate for the lower gluten content caused by the buckwheat. As more buckwheat is added, the gluten decreases, which can reduce the tensile strength of the noodles [52].

#### 3.3.4. Sensory Evaluation

Sensory evaluation helps measure how much consumers like a product. Table 7 shows how different pre-treatment methods of Tartary buckwheat flour affected noodle quality. When the flour made up 10% to 40%, the overall sensory scores increased and then decreased again. The best results came at 20% addition, with noodles displaying bright color, strong flavor, good taste, and the highest scores—no different from regular wheat noodles. Using pre-treated Tartary buckwheat instead of part of the wheat flour improves nutrition and also enhances flavor and texture. However, increasing the Tartary buckwheat content beyond 20% made the noodles darker, harder, and rougher on the surface, which lowered their sensory scores. At 20%, M-ETBF noodles had better taste and overall quality than ETBF noodles, making them more acceptable to consumers.

**Table 7:** Effect of pre-treatment Tartary buckwheat flour on the sensory quality of noodles.

Samples	Addition Amount/%	Color	External Appearance	Palatability (Softness and Hardness)	Tenacity	Stickiness	Smoothness	Taste	Total Score
ETBF	0	8.53 ± 0.94 ^a^	9.13 ± 0.54 ^a^	17.38 ± 0.82 ^a^	21.75 ± 1.30 ^a^	19.00 ± 2.35 ^a^	4.38 ± 0.65 ^a^	4.20 ± 0.74 ^a^	84.35 ± 2.06 ^a^
	10	9.07 ± 0.09 ^a^	8.63 ± 0.21 ^a^	14.33 ± 1.25 ^c^	17.00 ± 0.82 ^b^	18.33 ± 1.70 ^a^	4.17 ± 0.79 ^a^	4.37 ± 0.53 ^a^	75.90 ± 2.82 ^b^
	20	8.35 ± 0.27 ^a^	8.10 ± 0.70 ^a^	15.38 ± 0.96 ^bc^	20.25 ± 1.79 ^a^	19.00 ± 1.58 ^a^	4.43 ± 0.36 ^a^	4.60 ± 0.23 ^a^	80.10 ± 2.60 ^ab^
	30	6.20 ± 0.76 ^b^	6.40 ± 0.99 ^b^	16.50 ± 1.12 ^ab^	21.25 ± 0.83 ^a^	18.25 ± 0.83 ^a^	3.88 ± 0.72 ^a^	4.35 ± 0.41 ^a^	76.83 ± 3.70 ^b^
	40	3.23 ± 0.81 ^c^	6.33 ± 0.23 ^b^	16.00 ± 0.71 ^ab^	20.75 ± 0.83 ^a^	19.25 ± 1.79 ^a^	4.28 ± 0.57 ^a^	4.68 ± 0.41 ^a^	74.50 ± 3.68 ^b^
M- ETBF	0	8.53 ± 0.94 ^a^	9.13 ± 0.54 ^a^	17.38 ± 0.82 ^a^	21.75 ± 1.30 ^a^	19.00 ± 2.35 ^a^	4.38 ± 0.65 ^a^	4.20 ± 0.74 ^a^	84.35 ± 2.06 ^a^
	10	8.08 ± 0.72 ^a^	8.50 ± 0.87 ^a^	16.63 ± 2.10 ^a^	18.75 ± 2.86 ^ab^	18.63 ± 2.63 ^a^	4.08 ± 0.68 ^a^	4.45 ± 0.36 ^a^	79.10 ± 1.28 ^b^
	20	8.04 ± 0.52 ^a^	9.00 ± 0.50 ^a^	17.75 ± 0.83 ^a^	19.50 ± 1.12 ^a^	19.25 ± 1.30 ^a^	4.13 ± 0.74 ^a^	4.35 ± 0.55 ^a^	82.01 ± 3.50 ^ab^
	30	6.43 ± 0.90 ^b^	8.18 ± 0.78 ^a^	16.25 ± 1.79 ^a^	17.75 ± 3.83 ^ab^	16.75 ± 2.86 ^a^	3.90 ± 0.87 ^a^	3.80 ± 0.97 ^a^	73.05 ± 1.95 ^c^
	40	5.28 ± 0.91 ^b^	6.85 ± 0.92 ^b^	13.00 ± 1.58 ^b^	14.00 ± 3.24 ^b^	17.50 ± 3.64 ^a^	3.98 ± 0.81 ^a^	3.88 ± 0.89 ^a^	64.48 ± 3.66 ^d^

Notes: ETBF, extruded Tartary buckwheat flour; M-ETBF, microwave-extruded Tartary buckwheat flour. Different letters in the same column indicate significant differences (*p* < 0.05).

## 4. Conclusions

This study systematically compared the physicochemical properties of extruded Tartary buckwheat flour (ETBF) and microwave-extruded Tartary buckwheat flour (M-ETBF), and evaluated their effects on composite dough and fresh wet noodle quality. Significant differences were observed between the two pre-treated flours, which directly determined their processing performance and final product quality. In raw flour characteristics, M-ETBF exhibited 38.3% higher protein content and 33.4% higher total flavonoid content than ETBF. This nutritional advantage arises from microwave pre-treatment inhibiting rutin-degrading enzyme activity and promoting the formation of stable fat–starch–protein complexes, reducing nutrient loss during subsequent extrusion. For dough quality, increasing addition levels of both flours elevated gelatinization onset (T_0_) and peak (T_p_) temperatures, while decreasing gelatinization enthalpy (ΔH) and pasting viscosity. At the optimal 20% addition level, the color of the dough became darker, and the dough’s rheological properties approached those of wheat dough. M-ETBF dough showed longer midline peak time and larger midline peak area than ETBF dough, indicating superior kneading resistance and gluten network stability. Regarding noodle quality, both flours increased hardness and chewiness but decreased springiness with increasing addition. At equivalent addition levels, M-ETBF noodles consistently showed better textural properties at 20% addition, aligning with consumer preferences. M-ETBF noodles also had higher water absorption at all levels. Sensory evaluation revealed that 20% M-ETBF noodles achieved the highest overall score. In conclusion, microwave-extrusion combined pre-treatment is superior to single extrusion for Tartary buckwheat flour modification. M-ETBF retains more nutrients and imparts better processing adaptability and product quality. The optimal addition level of M-ETBF in fresh wet noodles is 20%, achieving a balance between nutritional enhancement and sensory quality.

## Figures and Tables

**Table 1:** Sensory scoring criteria for fresh wet Tartary buckwheat noodles.

Evaluation Attribute	Full Score	Scoring Criteria
Color	10	8.6–10: Bright and uniform color; 6.0–8.5: Moderate brightness, slightly uneven color distribution; 1.0–5.9: Dull, grayish color with highly uneven distribution.
Surface Appearance	10	8.6–10: Uniform and smooth surface structure; 6.0–8.5: Uniform surface structure with minor breakage; 1.0–5.9: Rough surface with severe deformation.
Firmness (Hardness/Softness)	20	17–20: Optimal firmness (proper bite force); 13–16: Slightly too firm or too soft; 1–12: Excessively firm or excessively soft.
Springiness & Chewiness	25	21–25: Excellent springiness and chewiness; 16–20: Moderate springiness and chewiness; 1–15: Poor springiness and chewiness.
Stickiness	25	21–25: Refreshing, non-sticky to teeth; 16–20: Slightly refreshing, mildly sticky to teeth; 1–15: Not refreshing, highly sticky to teeth.
Mouthfeel Smoothness	5	4.4–5: Smooth mouthfeel; 3.1–4.3: Moderately smooth mouthfeel; 1–3: Poor mouthfeel smoothness.
Flavor	5	4.4–5: Distinct Tartary buckwheat flavor, no off-flavors; 3.1–4.3: No obvious off-flavors; 1–3: Presence of off-flavors.

**Table 2:** Basic physicochemical elements of different Tartary buckwheat flours.

Samples	Moisture (%)	Protein (%)	Ash (%)	Fat (%)	Starch (%)	Total Flavonoid (mg RE/g)
ETBF	9.12 ± 0.43 ^a^	10.35 ± 0.05 ^b^	1.80 ± 0.01 ^b^	0.11 ± 0.02 ^b^	68.77 ± 0.47 ^a^	18.95 ± 0.20 ^b^
M-ETBF	9.32 ± 0.07 ^a^	14.30 ± 0.15 ^a^	2.27 ± 0.01 ^a^	1.10 ± 0.26 ^a^	68.12 ± 0.92 ^a^	25.27 ± 0.10 ^a^

Notes: ETBF, extruded Tartary buckwheat flour; M-ETBF, microwave-extruded Tartary buckwheat flour. Different letters in the same column indicate significant differences (*p* < 0.05).

**Table 3:** Color and particle size of different Tartary buckwheat flours.

Samples	Color Characteristics			Particle Size
	L*	a*	b*	Particle size/nm
ETBF	52.66 ± 0.03 ^b^	−2.11 ± 0.14 ^b^	27.44 ± 0.12 ^a^	833.32 ± 4.06 ^a^
M-ETBF	49.29 ± 0.04 ^c^	−2.04 ± 0.14 ^b^	27.22 ± 0.15 ^a^	625.75 ± 6.45 ^b^

Notes: L*, lightness; a*, red–green value; b*, yellow–blue value; ETBF, extruded Tartary buckwheat flour; M-ETBF, microwave-extruded Tartary buckwheat flour. Different letters in the same column indicate significant differences (*p* < 0.05).

**Table 6:** Effect of pre-treatment Tartary buckwheat flour on the kneading and mixing characteristics of dough.

Samples	Addition Amount/%	Midline Peak Time/min	Midline Peak Area/%Tq·min	The 8 min Bandwidth/%	Absolute Value of Right Slope
ETBF	0	2.08 ± 0.07 ^a^	87.04 ± 5.43 ^a^	10.02 ± 0.49 ^bc^	0.93 ± 0.82 ^c^
	10	1.68 ± 0.00 ^b^	67.17 ± 2.11 ^b^	17.71 ± 5.06 ^ab^	2.00 ± 0.43 ^c^
	20	1.39 ± 0.12 ^c^	55.60 ± 4.46 ^bc^	22.72 ± 0.91 ^a^	3.94 ± 0.51 ^b^
	30	1.25 ± 0.04 ^c^	51.58 ± 0.80 ^c^	20.07 ± 3.51 ^ab^	5.88 ± 0.34 ^a^
	40	1.18 ± 0.01 ^c^	46.69 ± 0.35 ^c^	4.00 ± 0.25 ^c^	7.44 ± 0.17 ^a^
M- ETBF	0	2.08 ± 0.07 ^a^	87.04 ± 5.43 ^a^	10.02 ± 0.49 ^b^	0.93 ± 0.82 ^c^
	10	1.97 ± 0.32 ^a^	81.66 ± 14.88 ^a^	23.90 ± 0.00 ^a^	1.54 ± 0.76 ^c^
	20	1.56 ± 0.34 ^ab^	60.05 ± 10.20 ^ab^	13.70 ± 0.70 ^b^	2.68 ± 0.75 ^bc^
	30	1.17 ± 0.02 ^b^	46.34 ± 1.45 ^b^	5.39 ± 2.51 ^c^	5.18 ± 0.37 ^b^
	40	1.16 ± 0.02 ^b^	47.93 ± 0.43 ^b^	4.37 ± 0.10 ^c^	8.16 ± 0.93 ^a^

Notes: ETBF, extruded Tartary buckwheat flour; M-ETBF, microwave-extruded Tartary buckwheat flour. Different letters in the same column indicate significant differences (*p* < 0.05).

## Data Availability

The data presented in this study are available in this article.

## References

[1] Krkošková B., Mrázová Z. (2005). Prophylactic components of buckwheat. Food Res. Int..

[2] Lee L.-S., Choi E.-J., Kim C.-H., Sung J.-M., Kim Y.-B., Seo D.-H., Choi H.-W., Choi Y.-S., Kum J.-S., Park J.-D. (2016). Contribution of flavonoids to the antioxidant properties of common and tartary buckwheat. J. Cereal Sci..

[3] Scalbert A., Manach C., Morand C., Rémésy C., Jiménez L. (2005). Dietary Polyphenols and the Prevention of Diseases. Crit. Rev. Food Sci. Nutr..

[4] Zheng C., Hu C., Ma X., Peng C., Zhang H., Qin L. (2012). Cytotoxic phenylpropanoid glycosides from *Fagopyrum tataricum* (L.) Gaertn. Food Chem..

[5] Bai Y.-P., Guo X.-N., Zhu K.-X., Zhou H.-M. (2017). Shelf-life extension of semi-dried buckwheat noodles by the combination of aqueous ozone treatment and modified atmosphere packaging. Food Chem..

[6] Mariotti M., Lucisano M., Ambrogina Pagani M., Ng P.K.W. (2009). The role of corn starch, amaranth flour, pea isolate, and Psyllium flour on the rheological properties and the ultrastructure of gluten-free doughs. Food Res. Int..

[7] Yalcin S. (2020). Quality characteristics, mineral contents and phenolic compounds of gluten free buckwheat noodles. J. Food Sci. Technol..

[8] Yoo J., Kim Y., Yoo S.-H., Inglett G.E., Lee S. (2012). Reduction of rutin loss in buckwheat noodles and their physicochemical characterisation. Food Chem..

[9] Sun X., Yu C., Fu M., Wu D., Gao C., Feng X., Cheng W., Shen X., Tang X. (2019). Extruded whole buckwheat noodles: Effects of processing variables on the degree of starch gelatinization, changes of nutritional components, cooking characteristics and in vitro starch digestibility. Food Funct..

[10] Sun X., Li W., Hu Y., Zhou X., Ji M., Yu D., Fujita K., Tatsumi E., Luan G. (2018). Comparison of pregelatinization methods on physicochemical, functional and structural properties of tartary buckwheat flour and noodle quality. J. Cereal Sci..

[11] Tian H.Y., Guo X.D., Li W.X., Ji X., Du L., Wang M. (2014). Study on the effects of different treatment temperatures on the contents of antioxidant components and antioxidant activity of tartary buckwheat. J. Chin. Cereals Oils Assoc..

[12] Bian Z.X., Wang J.F., Ma H., Wang S.M., Luo L., Wang S.M. (2020). Effect of microwave radiation on antioxidant capacities of Tartary buckwheat sprouts. J. Food Sci. Technol..

[13] Liu Y., Chen J., Luo S., Li C., Ye J., Liu C., Gilbert R.G. (2017). Physicochemical and structural properties of pregelatinized starch prepared by improved extrusion cooking technology. Carbohydr. Polym..

[14] Wang R., Li M., Chen S., Hui Y., Tang A., Wei Y. (2019). Effects of flour dynamic viscosity on the quality properties of buckwheat noodles. Carbohydr. Polym..

[15] Wang S., Wang J., Wang S., Wang S. (2017). Annealing improves paste viscosity and stability of starch. Food Hydrocoll..

[16] (2016). National Food Safety Standard—Determination of Moisture in Foods.

[17] (2016). National Food Safety Standard—Determination of Ash in Foods.

[18] (2016). National Food Safety Standard—Determination of Protein in Foods.

[19] (2023). Determination of Starch in Foods.

[20] (2016). National Food Safety Standard—Determination of Crude Fat in Foods.

[21] (2007). Determination of Flavones in Buckwheat and Its Products.

[22] Cheng W., Gao L., Wu D., Gao C., Meng L., Feng X., Tang X. (2020). Effect of improved extrusion cooking technology on structure, physiochemical and nutritional characteristics of physically modified buckwheat flour: Its potential use as food ingredients. LWT Food Sci. Technol..

[23] Zhang Z., Fan X., Zou L., Xing B., Zhu M., Yang X., Qin P. (2022). Phytochemical properties and health benefits of pregelatinized Tartary buckwheat flour under different extrusion conditions. Front. Nutr..

[24] He C., Zhang Z., Liu H., Gao J., Li Y., Wang M. (2018). Effect of rutin and quercetin on the physicochemical properties of Tartary buckwheat starch. Starch-Stärke.

[25] Cereals & Grains Association (2011). AACC Approved Method 54-40.01: Rheological Behavior of Flour by Mixograph (10-g Constant Flour Weight Procedure).

[26] Gao L., Cheng W., Fu M., Wu D., Tang X. (2022). Effect of improved extrusion cooking technology modified buckwheat flour on whole buckwheat dough and noodle quality. Food Struct..

[27] Li X.Q., Lv J.Y., Chen L., Dong L.Q., Chen Y.Y., Xu F. (2024). Effect of precooking time on the quality of noodles with different frozen storage time. LWT Food Sci. Technol..

[28] Zhang H., Fan M., Li Y., Wang L., Qian H. (2022). Study on the prediction model of basic components on the quality of buckwheat noodles. J. Texture Stud..

[29] Sharma P., Gujral H.S., Singh B. (2011). Antioxidant activity of barley as affected by extrusion cooking. Food Chem..

[30] Jozinović A., Šubarić D., Ačkar Đ., Babić J., Klarić I., Kopjar M., Lendić K.V. (2012). Influence of buckwheat and chestnut flour addition on properties of corn extrudates. Croat. J. Food Sci. Technol..

[31] Vicente A., Villanueva M., Caballero P.A., Muñoz J.M., Ronda F. (2023). Buckwheat grains treated with microwave radiation: Impact on the techno-functional, thermal, structural, and rheological properties of flour. Food Hydrocoll..

[32] Lee J., Kang Y.-R., Kim Y.J., Chang Y.H. (2019). Effect of high pressure and treatment time on nutraceuticals and antioxidant properties of *Lonicera japonica* Thunb. Innov. Food Sci. Emerg. Technol..

[33] Lund M.N., Ray C.A. (2017). Control of Maillard Reactions in Foods: Strategies and Chemical Mechanisms. J. Agric. Food Chem..

[34] Zou S., Sun Y., Li Z., Qiu J., Wang L. (2026). Effect of pre-gelatinization on the qualities of extruded whole Tartary buckwheat noodles. LWT.

[35] Žilić S., Mogol B.A., Akıllıoğlu G., Serpen A., Delić N., Gökmen V. (2014). Effects of extrusion, infrared and microwave processing on Maillard reaction products and phenolic compounds in soybean. J. Sci. Food Agric..

[36] Chakraborty I.N.P., Mal S.S., Paul U.C., Rahman M.H., Mazumder N. (2022). An insight into the gelatinization properties influencing the modified starches used in food industry: A review. Food Bioprocess Technol..

[37] Zhang Z., Zhu M., Xing B., Liang Y., Zou L., Li M., Qin P. (2023). Effects of extrusion on structural properties, physicochemical properties and in vitro starch digestibility of Tartary buckwheat flour. Food Hydrocoll..

[38] Jia F., Wang J., Wang Q., Zhang X., Chen D., Chen Y., Zhang C. (2020). Effect of extrusion on the polymerization of wheat glutenin and changes in the gluten network. J. Food Sci. Technol..

[39] Fan D., Wang L., Zhang N., Xiong L., Huang L., Zhao J., Zhang H. (2017). Full-time response of starch subjected to microwave heating. Sci. Rep..

[40] Zhang Z.J., Zhu F., Sun J., Li Y., Qian H., Wang L. (2021). Effects of microwave pretreatment and cooking on the digestive and physicochemical properties of red rice. Food Ferment. Ind..

[41] Peng Y., Chen J., Lv Y.G., Qu L. (2015). Effects of tartary buckwheat flour addition level on dough properties and steamed bread quality. Sci. Technol. Cereals Oils Foods.

[42] Han X.-M., Xing J.-J., Han C., Guo X.-N., Zhu K.-X. (2021). The effects of extruded endogenous starch on the processing properties of gluten-free Tartary buckwheat noodles. Carbohydr. Polym..

[43] Liu X.H., Han F., Zhu X., Wang C., Li H., Chen S. (2023). Properties of potato whole flour dough and preparation of tough biscuits. Cereals Oils.

[44] Lisovska T., Banaś K., Orkusz A., Harasym J. (2023). Hydrothermal treatment via microwave radiation improves viscoelastic properties of native gluten-free flours for extrusion 3D printing. Appl. Sci..

[45] Han C., Ma M., Li M., Sun Q. (2020). Further interpretation of the underlying causes of the strengthening effect of alkali on gluten and noodle quality: Studies on gluten, gliadin, and glutenin. Food Hydrocoll..

[46] Zhang M., Ma M., Yang T., Li M., Sun Q. (2022). Dynamic distribution and transition of gluten proteins during noodle processing. Food Hydrocoll..

[47] Fu M., Sun X., Wu D., Meng L., Feng X., Cheng W., Gao C., Yang Y., Shen X., Tang X. (2020). Effect of partial substitution of buckwheat on cooking characteristics, nutritional composition, and in vitro starch digestibility of extruded gluten-free rice noodles. LWT-Food Sci. Technol..

[48] Guo X.Y., Dong G.M., Shen R.L., Li Y.L. (2024). Effect of high-gelatinized tartary buckwheat whole flour on the quality of fresh wet noodles. J. Light Ind..

[49] Han C., Xing J.J., Guo X.N., Zhu K.X. (2022). Effects of extruded tartary buckwheat flour addition on the processing and quality of pure tartary buckwheat dried noodles. Food Ferment. Ind..

[50] Puligundla P., Lim S. (2021). Buckwheat noodles: Processing and quality enhancement. Food Sci. Biotechnol..

[51] Zou X., Wang X., Zhang M., Peng P., Ma Q., Hu X. (2023). Pre-baking-steaming of oat induces stronger macromolecular interactions and more resistant starch in oat-buckwheat noodle. Food Chem..

[52] Obadi M., Chen Y., Qi Y., Liu S., Xu B. (2020). Effects of different pre-gelatinized starch on the processing quality of high value-added Tartary buckwheat noodles. J. Food Meas. Charact..

